# Gluteal Muscle Fatty Atrophy: An Independent Risk Factor for Surgical Treatment in Elderly Patients Diagnosed with Type-III Fragility Fractures of the Pelvis

**DOI:** 10.3390/jcm12226966

**Published:** 2023-11-07

**Authors:** Christoph Linhart, Dirk Mehrens, Luca Maximilian Gellert, Christian Ehrnthaller, Johannes Gleich, Christopher Lampert, Maximilian Lerchenberger, Wolfgang Böcker, Carl Neuerburg, Yunjie Zhang

**Affiliations:** 1 Department of Orthopaedics and Trauma Surgery, Musculoskeletal University Center Munich (MUM), University Hospital, LMU Munich, 81377 Munich, Germany; christoph.linhart@med.uni-muenchen.de (C.L.); luca.gellert@med.uni-muenchen.de (L.M.G.); christian.ehrnthaller@med.uni-muenchen.de (C.E.); johannes.gleich@med.uni-muenchen.de (J.G.); christopher.lampert@med.uni-muenchen.de (C.L.); maximilian.lerchenberger@med.uni-muenchen.de (M.L.); direktion-unfall@med.uni-muenchen.de (W.B.); carl.neuerburg@med.uni-muenchen.de (C.N.); 2 Department of Radiology, University Hospital, LMU Munich, 81377 Munich, Germany; dirk.mehrens@med.uni-muenchen.de

**Keywords:** muscle fatty atrophy, FFP, geriatric trauma

## Abstract

Background: Gluteal muscle fatty atrophy (gMFA) might impair pelvic stability and negatively influence remobilization in patients with fragility fractures of the pelvis (FFP). This study aimed to investigate the association between gMFA and surgical indication in patients with FFP. Methods and materials: A retrospective analysis of 429 patients (age ≥80) diagnosed with FFP was performed. gMFA of the gluteus maximus, medius, and minimus was evaluated using a standard scoring system based on computer tomography images. Results: No significant difference was found in gMFA between genders or among FFP types. The severity of gMFA did not correlate with age. The severity of gMFA in the gluteus medius was significantly greater than in the gluteus maximus, whereas the most profound gMFA was found in the gluteus minimus. gMFA was significantly more severe in patients who underwent an operation than in conservatively treated patients with type-III FFP, and an independent correlation to surgical indication was found using logistic regression. Conclusion: Our findings imply that gMFA is an independent factor for surgical treatment in patients with type-III FFP. Besides focusing on the fracture pattern, the further evaluation of gMFA could be a feasible parameter for decision making toward either conservative or surgical treatment of type-III FFP.

## 1. Introduction

Fragility fractures of the pelvis (FFP) are amongst the most common low-energy traumatic fractures in elderly patients, particularly those over 80 years [1,2]. The three-year mortality in patients suffering from FFP was reported to be about 14%, and 17% in those hospitalized [3]. Pelvic fractures constitute 7% of all fractures related to osteoporosis in individuals aged over 50 in the United States, contributing to 5% of the overall cost burden [4]. The main goals of using either conservative or operative treatment in patients with FFP are to relieve pain and restore mobility and self-independency [5].

Muscle fatty atrophy (MFA), also known as muscle fat infiltration or myosteatosis, is characterized by fat accumulation within skeletal muscle tissue [6]. MFA tends to increase with age, contributing to a decline in muscle strength and function in older adults [7]. The biological mechanism of MFA is still unclear. The diagnosis of MFA often involves imaging techniques such as magnetic resonance imaging or computer tomography (CT), where the extent of fat infiltration within muscle tissue can be visualized [8].

The gluteal muscles, including the gluteus maximus, medius, and minimus, play a fundamental role in stabilizing the pelvis. They are the main stabilizers of the pelvis throughout the stride phase and facilitate the abduction and extension of the thigh [9,10]. They also contribute to balance, posture, and hip joint stability [11]. Daguet et al. reported that gluteal muscle fatty atrophy (gMFA) is associated with poorer physical performance, like chair rising [12]. The fatty atrophy of the gluteus minimus and medius might predispose the elderly to fall-related fractures [13]. The hip abductive strength was proven to be essential to the success of rehabilitation in hip-fractured patients [14,15]. Pelvic fractures like FFP disrupt the normal biomechanics of the pelvis. The atrophied gluteal muscle can result in difficulty with remobilization and pelvic instability in patients with FFP [13,16].

The surgical indication is partially dependent on the classification of the FFP according to the therapy recommendation by Rommens et al. [5,17,18]. A consensus about the optimal treatment regimen for patients with FFP has not been achieved. This is because the general condition of geriatric patients greatly affects the decision because their physiological reserve might be largely limited [19]. It is a multidisciplinary play between biomechanics and geriatric medicine. Fast remobilization could reduce complications such as deep venous thrombosis, pulmonary embolism, pressure ulcers, pneumonia, and decreased muscle strength [20]. Every extra day with immobilization in the hospital is accompanied by a drastically increased mortality rate. Consequently, the early identification of elderly patients with the indication for surgical therapy due to FFP might benefit remobilization and reduce the mortality rate.

Currently, the connection between MFA and fracture in geriatric patients has drawn more attention. Yerli et al. reported that fatty atrophy in the psoas muscle in elderly patients could influence the type of hip fractures [15]. Lee et al. demonstrated that the spinal fracture risk was increased by multifidus muscle fatty infiltration [21]. However, the influence of gMFA on the management of patients with FFP has rarely been discussed. Therefore, we aim to investigate the clinical significance of gMFA in elderly patients (age ≥ 80) with FFP. We hypothesized that gMFA was associated with a surgical indication in elderly patients with FFP. The clinical significance of the current study is the exploration of a potentially new factor, gMFA, which can be easily evaluated preoperatively from CT images so that surgical decisions can be made more accurately for elderly patients with FFP.

## 2. Methods and Materials

The current study protocol was approved and registered by the local ethics committee (Registration No. 518-18). The study was a retrospective observational single-center study. Patients (age ≥ 80) diagnosed with FFP and consecutively admitted to our university teaching hospital from January 2003 to December 2019 were enrolled. The exclusion criteria were pathological fracture, high-energy trauma, and patients with an established palliative concept. Informed consent was obtained from all study participants or their legal representatives. The patient data were retrieved from the inpatient database of our hospital and irreversibly anonymized before analysis in a confidential database (Microsoft Excel 2018, Microsoft Corporation, Redmond, WA, USA). Demographic data, including age and gender, were collected. Preoperative comorbidities, such as chronic kidney disease, cardiac insufficiency, and coronary artery disease, were collected.

Multidisciplinary geriatric co-management was carried out in our center for the clinical management of this patient group. A surgical indication was decided based on the fracture morphology/classification and, more importantly, on general conditions and basic illnesses that could have substantial effects on operative risk and outcomes of the patients, such as heart failure, kidney failure, or age. Surgeries were performed by a senior trauma surgeon in our center with minimally invasive techniques. Open reduction and internal fixations were performed only by necessity. If conservative treatment was indicated, patients received standardized pain management according to WHO (World Health Organization) guidelines. Physiotherapy was initiated as soon as possible with weight-bearing as-tolerated. An operation would be indicated and performed when early mobilization failed after 5 days. No follow-up was conducted after the surgery because it was considered irrelevant to the hypothesis.

The FFP classification used in the current study was first described by Rommens and Hofmann [17]. Briefly, type I was featured with an isolated anterior ring fracture. In type II, the posterior pelvic ring was fractured without displacement. Type III was characterized by unilateral fracture displacement on the posterior pelvic ring. When the fracture displacement was bilateral on the posterior pelvic ring, the fracture was rated as type IV. The classification was documented as the diagnosis of each patient and controlled by senior orthopedic and radiologic consultants.

Pelvic CT was performed for all patients as a clinical routine for pelvic fracture. Intravenous contrast fluid was administered if indicated. The CT scan included sections at least from the level of the lesser trochanter of the femur to the level of the iliac crest. The axial reconstruction of the pelvis at the level of the anterior inferior iliac spine was defined as a standard plane for scoring. This was because it provided a clear view of the muscle bellies of the gluteus maximus, medius, and minimus, which were approximately centered at this particular level. The coronal plane was used secondarily for evaluation if needed.

**Figure 1:**
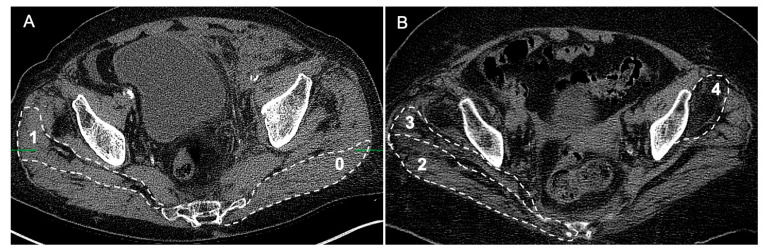
Examples of gMFA with different severities according to Goutallier grading scores based on CT pelvis images. (**A**) The area marked with 0 is an example of a score of 0 with no fat in the gluteus maximus, which was rare in the investigated population. The gluteus medius marked with 1 represented a score of 1 with intramuscular fatty steaks. (**B**) The area marked with 2 exemplified a score of 2 in gluteus maximus with mild atrophy, in which the proportion of fat infiltration was less than that of muscle. Nearby, an exaggerated fat infiltration could be found in the gluteus medius, marked by 3, where the proportion of fat and muscle were subjectively equal. The gluteus minimus, marked by 4, showed the most profound fat infiltration, where fat infiltration exceeded in quantity.

The classification system based on CT was first established by Goutallier et al. [22]. Score 0 was defined as normal muscle without obvious fatty infiltration, score 1 for minimal atrophy with minor fatty streaks, score 2 for mild atrophy with a lower volume of fatty infiltration than muscle, score 3 for moderate atrophy with an equal amount of fatty infiltration to the muscle, and score 4 for severe atrophy, by which the volume of fatty infiltration was greater than the volume of muscle tissue (Figure 1).

The primary scoring was performed by an orthopedic surgeon with three years of experience and then examined by an orthopedic surgeon with eight years of experience. Ambiguous cases were re-scored by consensus. Fifty cases were randomly selected and were rated separately by a musculoskeletal radiologist with six years of experience, and the intraclass correlation coefficient (ICC) was examined to ensure the reliability and reproducibility of the current method.

SPSS version 27.0 (SPSS Inc., Chicago, IL, USA) was used for statistical analysis. The ICC was calculated using Kendall’s tau method with ordinal data. For parametric data, the Kolmogorov–Smirnov test was performed to verify the normality. If normality was confirmed, a 2–sided t–test was used to determine the difference between the two groups; if not, the 2–sided Mann–Whitney U test was applied. For non-parametric data, the 2–sided Mann–Whitney U test was used. The Kruskal–Wallis test, followed by the Student–Newman–Keuls method, was performed to estimate stochastic probability in the intergroup comparison among three or more groups. The Pearson correlation coefficient (r) was used to identify the strength of the correlation. Multivariate logistic regression was performed to investigate the association of different potentially confounding variables such as sex, age, cardiac illness (heart failure or coronary artery disease), or renal insufficiency to the surgical treatment. A *p*-value of <0.05 was considered statistically significant.

## 3. Results

An overview of the included population in the current study is summarized in Table 1. A total of 429 patients ≥80 years diagnosed with FFP were investigated, from which the number of females was about 3.7 times the number of males. No difference was found in the rate of operation between genders. The rate of operation increased, confronting the severity of the fracture.

Kendall’s Tau test was performed to validate the reliability and reproducibility of the scoring method used in this study. Kendall’s coefficient value was 0.70 based on a subset of 50 randomly selected subjects, which was considered a strong interobserver agreement.

**Figure 2:**
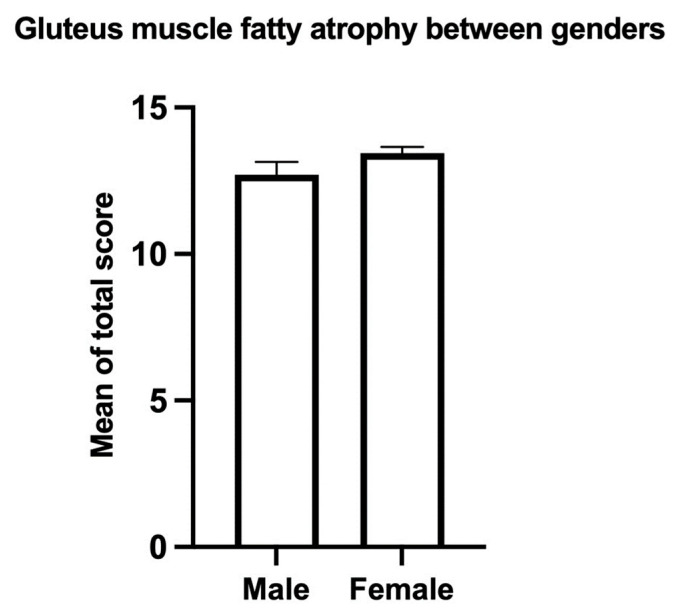
Mean comparison of gMFA using the total score between genders. Mean ± standard error of the mean. No significant difference was found in the overall score of gluteal fatty atrophy between genders.

**Figure 3:**
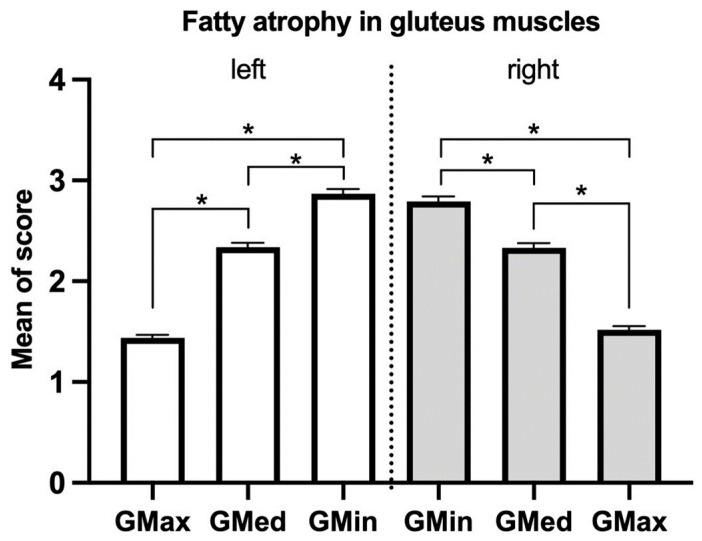
Average score of fatty atrophy in different gluteal muscles. Mean ± standard error of the mean. The score for fatty atrophy exhibited a significant increase in the gluteus medius compared to the gluteus maximus score. The most severe fatty atrophy was observed in the gluteus minimus, with no significant differences detected between the two sides. GMax: gluteus maximus, GMed: gluteus medius, GMin: gluteus minimus. * *p* < 0.001.

There was no significant difference in the total score of gMFA between genders (male 12.70 ± 4.27, female 13.44 ± 4.05, *p* = 0.23, Figure 2). The severity of each component of the gluteal muscles was evaluated (Figure 3). The score of gMFA was significantly higher (*p* < 0.001) in the gluteus medius (left: 2.34 ± 0.89, right 2.33 ± 0.97) compared to the score of gluteus maximus (left: 1.44 ± 0.65, right: 1.52 ± 0.76). The severest gMFA was found in the gluteus minimus (left: 2.87 ± 1.01, right: 2.79 ± 1.02, *p* < 0.001). No difference was found between the two sides.

**Figure 4:**
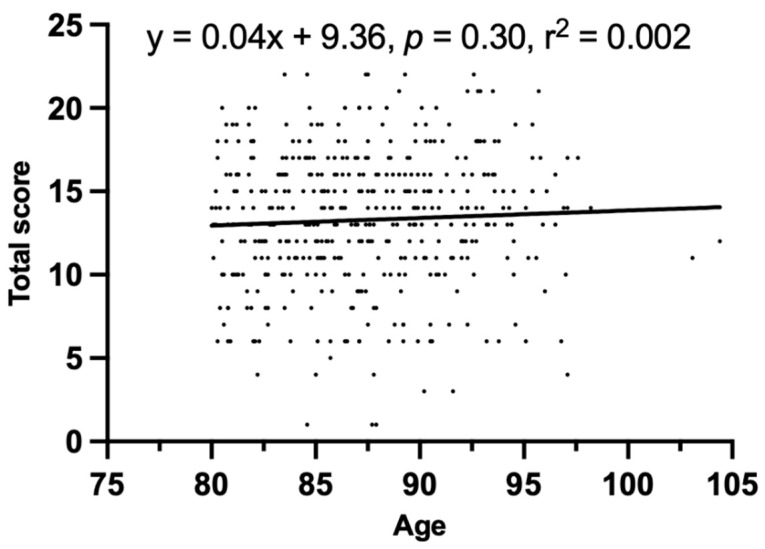
Correlation between age and gMFA.

**Figure 5:**
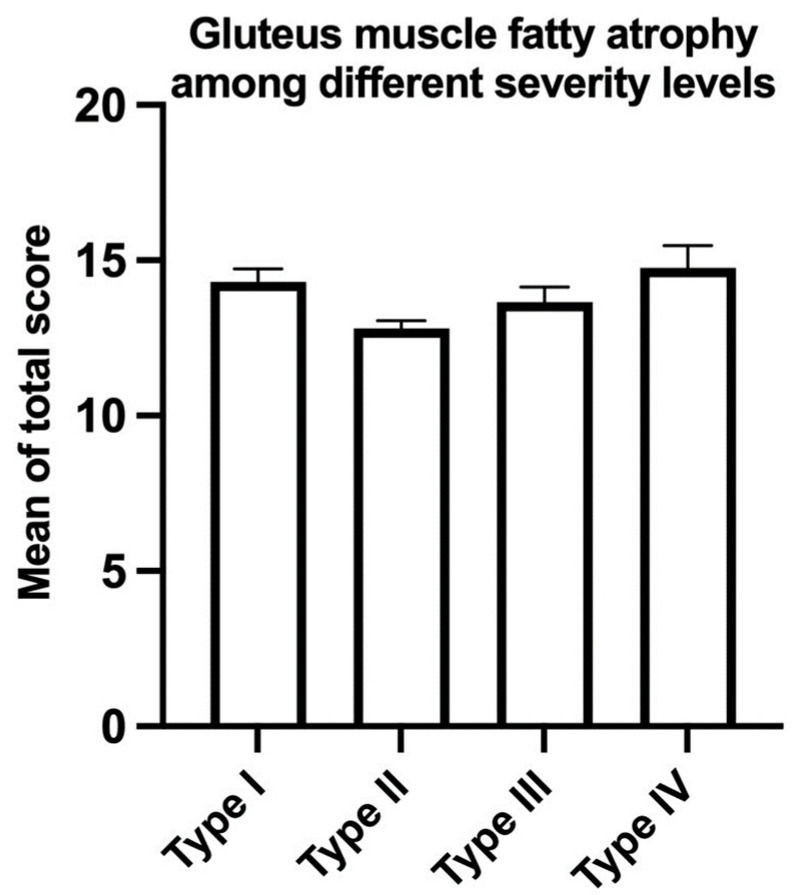
Mean comparison of gMFA using the total score among different FFP classifications. Mean ± standard error of the mean. There was no significant intraclass difference in the total fatty atrophy score observed across type-I to type-IV FFP.

The correlation between age and gMFA in patients over 80 with FFP was analyzed, and no significant correlation was found (y = 0.04 x + 9.36, *p* = 0.30, r^2^ = 0.002, Figure 4). No significant intraclass difference in the total fatty atrophy score was found among type-I to type-IV FFP (Figure 5).

**Figure 6:**
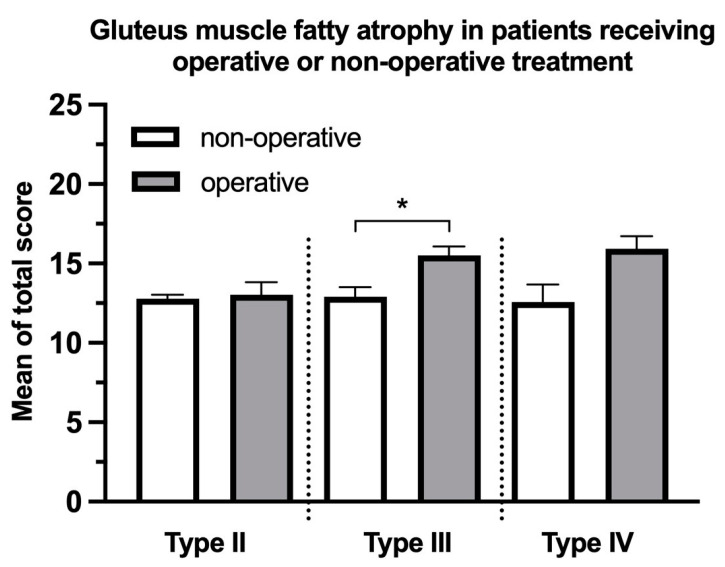
Mean comparison of gMFA using the total score between patients receiving operation and conservatively treated patients in type-II, type-III, and type-IV FFP. Mean ± standard error of the mean. Patients diagnosed with type-III FFP who underwent operative treatment exhibited significantly more pronounced gluteal fatty atrophy than those undergoing non-operative treatment. * *p* < 0.05. The analysis of type I was not performed because there was only 1 case operated.

The gMFA was significantly more severe in patients diagnosed with type-III FFP receiving operative treatment than those receiving non-operative treatment (15.50 ± 2.61 and 12.90 ± 4.25, respectively, *p* = 0.01, Figure 6). The mean total score of gMFA in the patients receiving surgery was higher than those receiving non-operative treatment diagnosed with type-II (13.03 ± 4.27 and 12.78 ± 4.12, respectively, *p* = 0.80) and type-IV FFP (15.92 ± 2.90 and 12.57 ± 2.94, respectively, *p* = 0.06) though both were without statistical significance. The analysis of type I was not performed because there was only one operated case.

Further, logistic regression was conducted to evaluate the association between gMFA and operative decisions in patients with type-III FFP. The total score of gMFA was significantly associated with the operative decision when other factors that could influence operability, such as age, cardiac illness (heart failure or coronary artery disease), or renal insufficiency, were also included (OR: 1.22, 95% CI: 1.01–1.47, *p* < 0.05, Table 2).

## 4. Discussion

The key finding of the current study is that the severity of gMFA could potentially be a factor for surgical indication in geriatric patients with type-III FFP. The early determination of potential surgical candidates with the help of possible factors, independent from fracture classification in orthogeriatric patients with FFP, could prompt the remobilization and reduce complications due to immobilization and prolonged hospital stays.

So far, there is no gold standard for the treatment of patients with FFP. Surgical treatments are often first indicated after the failure of conservative treatment due to the patient’s frailty, causing a relatively higher intraoperative and postoperative risk for bleeding, postoperative infection, postoperative delirium, and cardiovascular and renal complications [23]. A steep increase in incidence and economic burden caused by FFP in the elderly population, especially those above the age of 80, has been reported in many countries like the USA, Germany, and Finland [1,4,24]. This is associated with demographic change, leading to a growth in the geriatric population [25]. To the author’s knowledge, the current study is the first effort to discuss the impact of gMFA on the management of very elderly patients with FFP.

Aging is recognized as an important factor for gMFA [26]. However, the positive correlation between aging and gMFA did not exist in the current study population, where all patients were older than 80. Our result showed that the biological status of the muscle did not match the real age in this patient group. One of our previous works found that the speed of postoperative remobilization in the geriatric population with pertrochanteric fracture was significantly quicker in patients with better nutrition status and was also independent of age [27]. This finding implies that age is no longer a reliable reference in managing a very elderly population. A more comprehensive evaluation should be given to determine the operability and patient’s potential for remobilization.

In addition, unlike the incidence of osteoporosis, which was reported to be higher in females, the scale of gMFA did not differ between genders. No difference in gMFA was found among the four types of FFP. Thus, the severity of gMFA was not associated with the fracture complexity induced by trauma. Furthermore, the severity of gMFA was unequal among the gluteus maximus, medius, and minimus and was significantly more profound in the gluteus medius and minimus. This is possibly due to a decreased demand for hip abduction, supported by gluteus medius and minimus, in the daily activity of the elderly population. By contrast, the hip extension supported by the gluteus maximus is more frequently used, for example, in rising from chairs and stair climbing. Physiologically, the gluteus medius and minimus are critically involved during the stride to support the pelvic balance. Fatty atrophy in these two muscles could impair the mobility of the elderly in daily life and also remobilization after fractures like FFP [28,29]. Chi et al. reported that the gluteus medius and minimus fatty atrophy are associated with fall-related fractures [13]. These findings implied that functional training and active treatment due to the injury of gluteal muscle, especially gluteus medius and minimus, could be important for fall prevention and remobilization after a pelvic fracture [30].

Interestingly, the current study found that the total score of gMFA in patients undergoing surgeries with type-III FFP was significantly greater than in non-operated patients. The posterior pelvic ring in type-III FFP was completely interrupted with dislocation. In this case, the additional stability provided by the gluteal muscle might be critical for the patient’s mobilization after a fracture. Meanwhile, gMFA decreased muscle strength and impaired muscle function due to disrupting the normal contractile function of muscle fibers [31,32]. Patients suffering from type-III FFP with preclinical severe gMFA had generally weakened gluteal muscle function, which could negatively influence their baseline mobility. Our results indicate that these patients are more likely to need surgery to help them achieve remobilization. In the current patient group (age ≥ 80), comorbidities could also influence the operation decision. Logistic regression showed that gMFA is an independent factor associated with surgical treatment.

By contrast, gMFA played a less important role in FFP type II and IV. Although the total score was slightly higher, no significance was found in patients receiving surgical treatment compared with those conservatively treated. According to the classification and the anatomy, the bony posterior pelvic structure in type-II FFP was fractured but not dislocated with or without an anterior fracture. Being the largest subgroup with 289 patients, only about 10% of patients diagnosed with type-II FFP underwent an operation. In other words, type-II FFP is considered relatively stable, so the loss of muscle function seems less decisive in maintaining stability and does not affect the surgical indication. On the other hand, 20 patients with type-IV FFP were concluded in the current study with a 65% operation rate. This subtype of FFP was characterized by marked vertical instability on both sides of the posterior pelvis, for which surgical treatment was strongly recommended due to its instability [5]. In this context, the surgical indication is mainly induced by the high instability of the fracture. The gMFA, contrarily, might have only a limited impact.

Certain limitations must be recognized in the current study. First, it was a single-center retrospective study. The study design was controlled by using the STROBE checklist for observational study [33] (Supplementary Materials). The surgical indication might vary largely from center to center. Our hospital is a geriatric traumatology center. Working together with geriatrics, the surgical indication for these patients was decided in consensus and should be representative. The sample size for type-I, II, and IV FFP was relatively small due to the uneven distribution of the fracture types and, more importantly, the age threshold (≥ 80) of inclusion criteria. This caused limitations in the variables chosen to build the logistic regression model. Consequently, a multi-center study with more study objects should be conducted to examine the current hypotheses. Moreover, other factors like osteoporosis can also be associated with the tendency of surgical treatment. It would be interesting to include bone mineral density test results and to conduct future studies from a biomechanical perspective.

Taken together, gMFA was commonly found in very elderly patients with FFP without gender differences. The severity of gMFA did not correlate with age and was more profound in gluteus minimus and medius than maximus in those orthogeriatric patients. The gMFA was an independent factor for surgical indication in patients with type-III FFP.

## Figures and Tables

**Table 1:** Baseline clinical data.

			n = 429	%	Operation, n	%	Age, Mean ± SD
Gender	Male		91	21.21%	14	15.38%	86.85 ± 4.49
	Female		338	78.79%	49	14.50%	87.42 ± 4.54
Classification	I		55		1	1.82%	87.60 ± 4.02
		Ia	52	94.55%	0	0.00%	87.77 ± 4.00
		Ib	3	5.45%	1	33.33%	84.70 ± 3.92
	II		286		29	10.14%	87.16 ± 4.45
		IIa	56	19.65%	4	7.14%	86.78 ± 5.68
		IIb	37	12.98%	6	16.22%	86.64 ± 4.37
		IIc	193	67.72%	19	9.84%	87.36 ± 4.25
	III		68		20	29.41%	88.26 ± 4.95
		IIIa	14	19.72%	1	7.14%	89.85 ± 6.09
		IIIb	22	30.99%	8	36.36%	88.24 ± 4.23
		IIIc	32	45.07%	11	34.38%	87.57 ± 4.87
	IV		20		13	65.00%	85.78 ± 5.19
		IVa	7	33.33%	4	57.14%	88.49 ± 6.22
		IVb	6	28.57%	5	83.33%	83.41 ± 2.64
		IVc	7	33.33%	4	57.14%	85.11 ± 5.00

SD: standard deviation.

**Table 2:** Analysis of risk factors for operative indication in patients diagnosed with type-III FFP using logistic regression.

Factors	OR	95% CI	*p*-Value
Sex (male)	1.13	0.27–4.78	0.87
Age	0.93	0.83–1.05	0.26
Total score	1.22	1.01–1.47	0.04 *
HF or CAD	0.63	0.12–3.31	0.39
CKD	1.90	0.42–8.53	0.11

OR: odds ratio; CI: confidence interval; HF: heart failure; CAD: coronary artery disease; CKD: chronic kidney disease. * *p* < 0.05.

## Data Availability

The data presented in this study are available on request from the corresponding author.

## Supplementary Materials

The following supporting information can be downloaded at: https://www.mdpi.com/article/10.3390/jcm12226966/s1, Figure S1: STROBE Statement—Checklist of items that should be included in reports of cohort studies.
